# Three-Dimensional Structure of TspO by Electron Cryomicroscopy of Helical Crystals

**DOI:** 10.1016/j.str.2010.03.001

**Published:** 2010-06-09

**Authors:** Vladimir M. Korkhov, Carsten Sachse, Judith M. Short, Christopher G. Tate

**Affiliations:** 1 Medical Research Council Laboratory of Molecular Biology, Hills Road, Cambridge CB2 0QH, UK

**Keywords:** PROTEINS, CELLBIO

## Abstract

The 18 kDa TSPO protein is a polytopic mitochondrial outer membrane protein involved in a wide range of physiological functions and pathologies, including neurodegeneration and cancer. The pharmacology of TSPO has been extensively studied, but little is known about its biochemistry, oligomeric state, and structure. We have expressed, purified, and characterized a homologous protein, TspO from *Rhodobacter sphaeroides*, and reconstituted it as helical crystals. Using electron cryomicroscopy and single-particle helical reconstruction, we have determined a three-dimensional structure of TspO at 10 Å resolution. The structure suggests that monomeric TspO comprises five transmembrane α helices that form a homodimer, which is consistent with the dimeric state observed in detergent solution. Furthermore, the arrangement of transmembrane domains of individual TspO subunits indicates a possibility of two substrate translocation pathways per dimer. The structure provides the first insight into the molecular architecture of TSPO/PBR protein family that will serve as a framework for future studies.

## Highlights

► *R. sphaeroides* TspO reconstituted as helical tubular crystals ► Cryo-EM and image analysis lead to structure of TspO at 10 Å resolution ► The structure confirms a dimeric five transmembrane domain architecture of TspO ► Two putative substrate translocation pathways per dimer are suggested

## Introduction

The human 18 kDa translocator protein (TSPO), formerly known as the peripheral benzodiazepine receptor (Papadopoulos et al., 2006a), is involved in a wide range of physiological functions and pathological conditions. TSPO is also among the key targets of Valium (diazepam), which is one of the most frequently prescribed drugs for anxiety disorders (Braestrup et al., 1977, Gavish et al., 1999). Among the established molecular functions of TSPO are translocation of cholesterol (Li and Papadopoulos, 1998, Li et al., 2001) and porphyrins (Taketani et al., 1994) across the mitochondrial outer membrane, where a large fraction of TSPO is normally localized within the cell. On a cellular level, TSPO has been shown to be involved in steroid biosynthesis (Lacapere and Papadopoulos, 2003), cellular respiration (O'Hara et al., 2003), proliferation (Galiegue et al., 2004), and apoptosis (Maaser et al., 2001). In addition, biochemical and pharmacological data have implicated TSPO in a wide range of pathological conditions, including epilepsy (Veenman et al., 2002), neurodegenerative diseases (Papadopoulos et al., 2006b), and cancer (Han et al., 2003, Maaser et al., 2002, Weisinger et al., 2004). TSPO is also an established positron emission tomography marker for pathologies of the central nervous system (Zhang et al., 2004). A wealth of pharmacological data on TSPO has been published and high-affinity ligands have been developed (Papadopoulos et al., 2006a, Scarf et al., 2009). However, although attempts have been made to study the purified and reconstituted TSPO (Delavoie et al., 2003, Garnier et al., 1994, Lacapere et al., 2001), little is known about its three-dimensional (3D) structure, oligomeric state, and mode of action, i.e., whether it operates as a pump, a transporter, or a channel (Papadopoulos et al., 2006a). Bioinformatic predictions and hydropathy analyses, confirmed by biochemical and biophysical evidence, have indicated that TSPO and its bacterial homologs probably contain five α-helical transmembrane domains that possibly form dimers or multimers (Bogan et al., 2007, Papadopoulos et al., 1994, Yeliseev and Kaplan, 2000).

Structural studies of eukaryotic membrane proteins are still difficult. Expressing, purifying, and stabilizing eukaryotic membrane proteins presents formidable obstacles that need to be overcome before their structures can be determined by X-ray crystallography, electron cryomicroscopy (cryo-EM), or nuclear magnetic resonance (NMR). To obtain insight into the molecular structure of the proteins belonging to the TSPO family, we have therefore used a bacterial homolog, tryptophan-rich sensory protein (TspO) from *Rhodobacter sphaeroides*, as a structural and functional model of the human protein.

**Figure 1:**
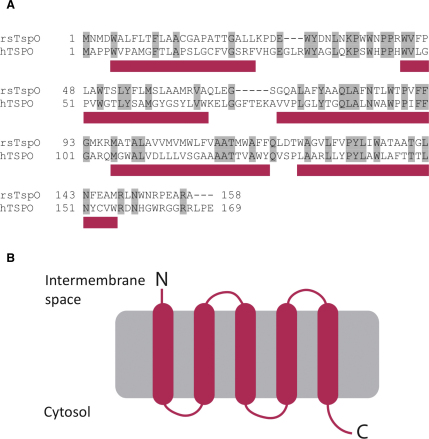
Comparison of Sequence and Topology of the Human and Bacterial TspO Homologues (A) Alignment between the amino acid sequences of human TSPO (hTSPO) and TspO from R. sphaeroides (rsTspO). Bars underneath the sequences represent hydrophobic regions that are predicted by hydropathy analysis to form transmembrane domains. (B) Cartoon of the predicted topology of *R. sphaeroides* TspO based on hydropathy analysis and the “positive inside” rule.

TspO from *R. sphaeroides* is involved in the control of photosynthetic gene expression and it has been proposed to act as an oxygen sensor (Yeliseev et al., 1997) that downregulates the photopigment genes in response to oxygen (Yeliseev and Kaplan, 1995, Yeliseev and Kaplan, 1999, Zeng and Kaplan, 2001). Its primary structure is very similar to that of the human TSPO, with 33.5% identity between the aligned amino acid sequences of *R. sphaeroides* TspO and human TSPO (Figure 1), excluding the longer interhelical loops in TSPO (Yeliseev and Kaplan, 1995); this level of homology is highly significant. Furthermore, bacterial TspO has been suggested to have functional and structural similarities to the human protein (Yeliseev and Kaplan, 2000), making it an ideal candidate for biochemical and structural work. Here we report the structure of *R. sphaeroides* TspO at 10 Å resolution, which represents the first structural data for a member of this family.

## Results

### Expression and Purification of TspO in *Escherichia coli*

**Figure 2:**
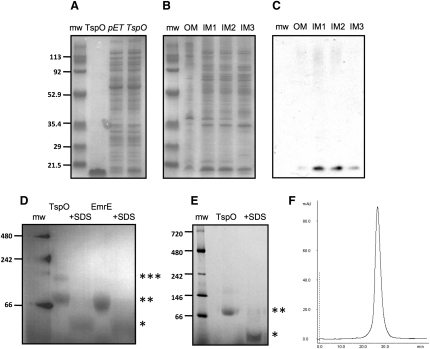
Dimeric TspO Is Expressed in the *E.* coli Inner Membrane (A) TspO protein expression was confirmed by Coomassie blue staining of the SDS-PAGE gel. TspO was enriched in a small-scale batch NiNTA purification procedure in DDM (lane *TspO*) and compared with control cells (lane *pET*). DDM-purified TspO is shown for comparison (lane TspO). (B and C) Fractionation of the TspO-expressing BL21(DE3) cell membranes. Lane OM corresponds to the outer membrane; lanes IM1–IM3 correspond to the inner membrane band (IM2) and the flanking fractions IM1 and IM3 that also contained inner membrane material. The gel in (B) was stained with Coomassie blue, whereas (C) is a western blot of an identical gel probed with an anti-poly His antibody to identify the position of recombinant TspO. (D and E) Blue native PAGE of purified TspO and purified EmrE. The asterisk indicates the monomeric species formed upon addition of 1% SDS for 10 min prior to the addition of the loading dye (+SDS). As a control, a standard dimeric membrane protein of a comparable size (15.2 kDa including the C-terminal tag), EmrE, is shown. Two and three asterisks indicate dimeric and multimeric protein species, respectively. If TspO is concentrated to below 5 mg/ml, the multimeric band is negligible (E). (F) Size exclusion chromatography profile of TspO in DDM.

TspO from *R. sphaeroides* was expressed in *E. coli* strain BL21(DE3) (Figure 2A). Contrary to the reports of TspO localization in the outer membrane of *R. sphaeroides*, the recombinantly expressed protein was inserted predominantly into the *E. coli* inner membrane (Figure 2B and 2C). This is unsurprising, given the predicted helical nature of TspO and the fact that all known bacterial outer membrane proteins have been shown to be β-barrel proteins, with the exception of Wza that contains a single C-terminal transmembrane α helix (Dong et al., 2006). TspO was expressed with a C-terminal His_6_ tag, which allowed its purification in dodecylmaltoside (DDM) by Ni^2+^-affinity chromatography and preparative-scale size exclusion chromatography. The purified TspO was monodisperse by size exclusion chromatography (Figure 2F) and was highly pure on Coomassie blue-stained SDS polyacrylamide gels (Figure 2A).

### Detergent-Solubilized TspO Is Dimeric

TspO purified in DDM was subjected to blue native PAGE analysis (Figures 2D and 2E). TspO formed two bands on the gel, with the predominant species appearing at an apparent molecular weight of ∼80 kDa and a minor species at an apparent molecular weight of ∼160 kDa. As these sizes include the mass of bound detergent, lipids, and Coomassie blue, it is not possible to use these data directly to determine the oligomeric state of TspO. However, when TspO was incubated in 1% SDS, the apparent mass decreased, which is consistent with the disruption of an oligomeric species by the harsh detergent. For comparison, EmrE was used as a control because it is known to be a homodimeric membrane protein (Butler et al., 2004) of comparable size (15 kDa in its tagged form) to TspO (18 kDa); the mobility of TspO in the blue native gels in the presence or absence of SDS is virtually identical to EmrE. All these data are consistent with TspO purified in DDM being a dimer. The variable appearance of a higher molecular weight band in the TspO sample was likely due to protein aggregation during its concentration because the higher molecular weight fraction was negligible when the concentration of purified TspO was kept below 5 mg/ml (Figure 2E). This is consistent with size exclusion chromatography of TspO purified in DDM showing a monodisperse protein peak (Figure 2F) and is similar to the behavior of mouse TSPO upon analysis by blue native PAGE (Rone et al., 2009).

### Purified TspO in Detergent Binds Porphyrins

**Figure 3:**
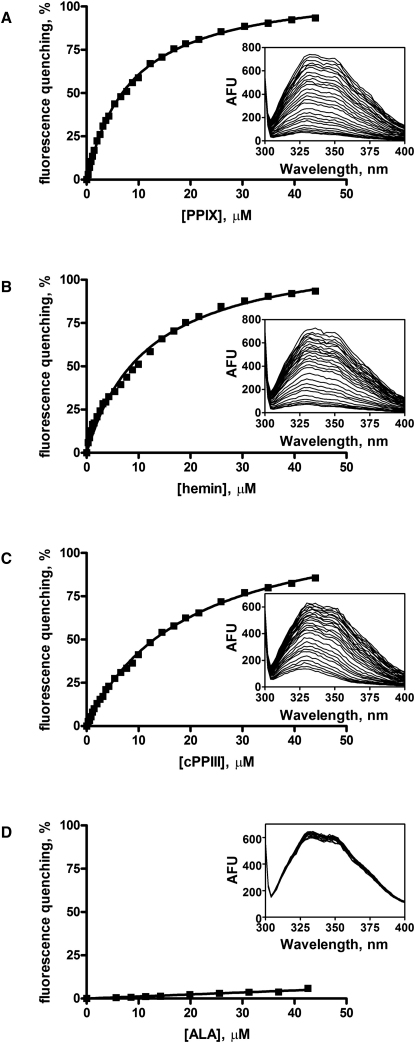
Binding of Substrates to TspO TspO purified in DDM was titrated with increasing amounts of PPIX (A), hemin (B), and cPPIII (C) and the tryptophan fluorescence was measured at 340 nm. For comparison, the measurements using the nontransported molecule D-aminolevulinic acid (ALA) is shown in (D). The tryptophan fluorescence emitted at 340 nm was plotted against the concentration of the quenching reagent to obtain a dose-response curve. The insets in each graph show the raw spectra in the 300 to 400 nm range. The data shown are the representative dose-dependence profiles of the tryptophan fluorescence quenching by the indicated substances; the number of experiments for PPIX, hemin, and cPPIII were three, two, and two, respectively.

To establish the functional integrity of the expressed and purified TspO, we first performed in vivo and in vitro uptake and binding assays on either intact *E. coli* or membrane preparations, respectively, both containing overexpressed TspO. Although the results of these experiments were indicative of an ability of the expressed TspO to bind and transport porphyrins, there was very high background binding to control cells not expressing TspO, probably because of the hydrophobicity of porphyrins, precluding accurate determination of substrate transport and binding affinity constants (data not shown). Therefore, intrinsic tryptophan fluorescence quenching was used to monitor porphyrin binding to purified TspO (Figure 3). We tested the binding of three different porphyrin analogs to purified TspO, namely, protoporphyrin IX (PPIX), hemin, and coproporphyrin III (cPPIII). Titration of TspO with increasing ligand concentrations revealed an efficient fluorescence quenching by all three analogs (Figures 3A–3C), with apparent half-maximal binding constants, K_app_, of 8.6 ± 0.6 μM for PPIX, 10.0 ± 2.5 μM for hemin, and 17.9 ± 1.7 μM for cPPIII (the values are mean ± SD). All three porphyrin analogs interact with TspO with similar affinities, which suggests that TspO may have a broad range of porphyrin specificity in vivo. These data confirmed that TspO was expressed and purified in a functional state. Moreover, the observed values are comparable to the reported affinity of PPIX for mouse TSPO, with a K_i_ of 18.95 μM (Wendler et al., 2003). No fluorescence quenching was observed when the nontransported molecule D-aminolevulinic acid was added to TspO (Figure 3D).

### TspO Reconstitution Yields Tubular Helical Crystals

**Figure 4:**
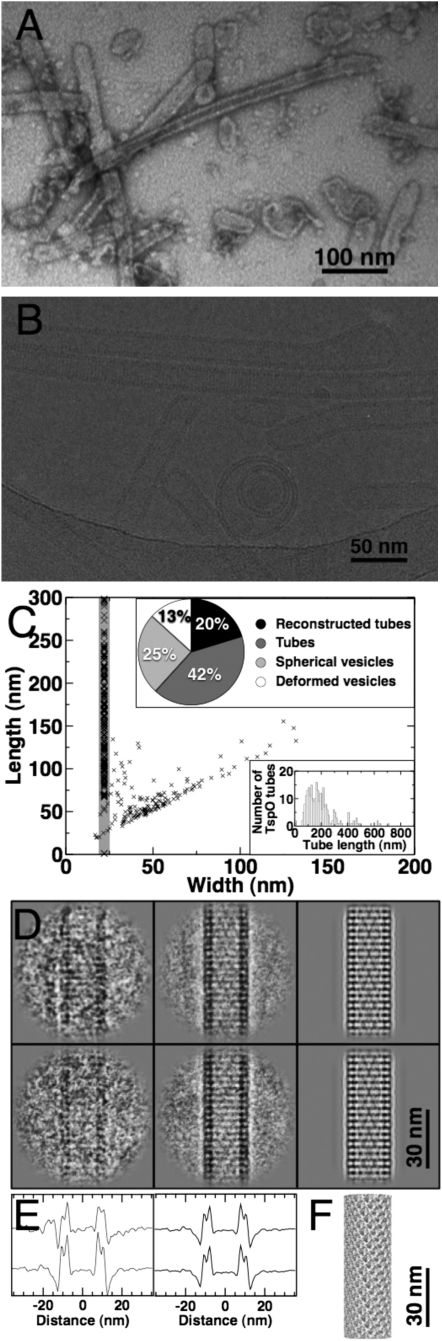
Cryo-EM Analysis of the TspO Helical Crystals (A) An image of negatively stained TspO tubes and small vesicles formed on TspO reconstitution; the scale bar corresponds to 100 nm. (B) Cryo-EM of vitrified TspO tubes on a holey carbon grid. The stacked disk arrangement of the well-preserved tubes is evident from unprocessed images; the scale bar corresponds to 50 nm. (C) Scatter plot of length and width of lipid structures present in the ice-embedded sample. The 25 nm wide tubes constitute two thirds of the sample and vesicles make up the remainder as shown in the pie chart (top inset). The distribution of tube lengths ranged from 100 to 1000 nm (bottom inset). (D) Comparison of an unprocessed cryo-EM image of a tube segment (left) with an average of the aligned image segments of the same azimuthal views (center) and the matched projection from the 3D image reconstruction, which was convoluted by its corresponding CTF (right). The top and bottom row differ in their azimuthal view by a rotation of 16° about the tube axis. (E) Width profiles correspond to the images displayed above (left and center). The pixel rows perpendicular to the tube axis were added to yield an averaged width profile of the tubes, which shows that all included segments contain tubes with a width of 25 nm (left). Averaged projection classes give rise to a less noisy profile (center). (F) Three-dimensional surface presentation of a TspO segment reconstructed at 10 Å resolution.

Purified TspO was dialyzed in the presence of purified lipids in attempts to produce well-ordered 2D crystals for cryo-EM. A systematic screen of the reconstitution conditions with varying lipids, lipid-to-protein ratios, buffer composition, temperature, and dialysis time was performed, which led to conditions where TspO formed narrow tubular crystals as observed by electron microscopy of negatively stained specimens (Figure 4A). TspO tubes were rapidly frozen on holey carbon grids by plunging them into liquid ethane and cryo-EM images were collected under low-dose conditions at liquid nitrogen temperature. The ice-embedded tubes were 100–1000 nm long and 25 nm in diameter (Figure 4C). The tubes make up 62% of lipid structures in the sample with another quarter belonging to a group of spherical vesicles. The remaining 13% of lipid structures consisted of deformed elliptical vesicles that may represent intermediate stages of partially assembled crystals (Figure 4C, inset).

### Three-Dimensional Reconstruction and Molecular Architecture of TspO in the Membrane

To obtain 3D structural information of TspO, tubes were analyzed by cryo-EM and a 10 Å resolution map was determined using single-particle helical reconstruction. The micrographs of well-preserved tubes showed a clearly distinguishable stacked disk-like morphology (Figure 4D). From 36 micrographs, a total of 205 straight tubes were segmented into 7542 overlapping image squares of an 108 nm dimension. Initially, the principal helical organization of the tubes could be derived from Fourier analysis of averaged tube images (see Figures S1B and S1C available online).

To ensure that all the processed tubes consisted of the same helical packing, we used multivariate statistical analysis to classify tube segments to carefully analyze their width and the helical diffraction pattern. First, the width of all individual segments and their class averages was determined to be 25 nm (Figure 4E). Second, two out of twelve classes gave rise to an interpretable set of three identical layer lines (Figure S1B). Subsequent indexing allowed the building of the helical lattice (Figures S1C and S1D; see Experimental Procedures). Hence, the tubes were composed of arrays of stacked rings each consisting of 12 TspO dimers, each disk being 32.7 Å thick and related to the neighboring disk by a rotation of 9.5° about the helical axis (Figure S1D). Subsequently, the derived helical symmetry parameters were refined using single-particle processing (Figures S1F and S1G) (Low et al., 2009). For the 3D image reconstruction, we used a recently developed single particle-based method that exploits helical symmetry (Sachse et al., 2007). To further include only those segments with a visible stacking order, we excluded two thirds of tube segments that were devoid of the 32 Å layer line diffraction; 2610 segments, i.e., 20% of the tube images, were included in the final density map at 10 Å resolution (Figures 4D and 4F; Figure S3) (the statistics of the reconstruction are shown in Table 1).

**Table 1:** Statistics of Helical Reconstruction

Image Processing Statistics	
Resolution FSC at 0.5/0.143 (Å)	10.2/7.8
Total length of nonoverlapping segments (nm)	38,894
Number of tubes	205
Number of segments/finally included	7,542/2,610
Segment size (nm)	108
Size of 3D reconstruction (nm)	72
Segment step size (nm)	3.2
Helical rise (Å)	32.67
Helical rotation (°)	9.53
Pixel size on the specimen (Å)	1.20
Number of asymmetric units	62,640

**Figure 5:**
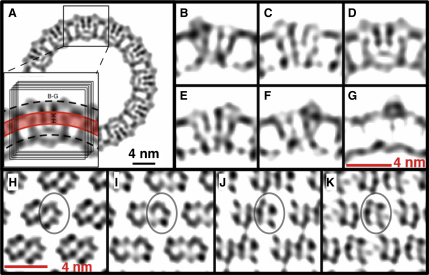
Grayscale Density Slices of the TspO 3D Reconstruction (A) A slice of density through the helical tube with the inset depicting the orientation of the 4.8 Å thick slices depicted in (B)–(K). (B–G) TspO viewed parallel to the membrane plane; slices of density were made perpendicular to the helical axis of the tube, at various distances from the center of the dimer (D), including −9.6 Å (B), −4.8 Å (C), 4.8 Å (E), 9.6 Å (F), and 14.4 Å (G). (H–K) The TspO dimer viewed perpendicular to the membrane plane; radial slices of density were taken through the membrane plane at positions −7.2 Å (H), −2.4 Å (I), +2.4 Å (J), and +7.2 Å (K) with respect to the center of the bilayer, with + being toward the external surface of the tube. The density interpreted as one molecule of TspO has been circled.

The resulting EM model of TspO revealed a molecular arrangement in which a pair of TspO monomers form a tightly associated symmetrical dimer of approximate dimensions 40 × 27 Å in the membrane plane and about 40 Å perpendicular to the membrane (Figure 5). The cross-sections through the final density map perpendicular to the lipid bilayer plane reveal low-density areas formed within the individual TspO monomers (Figures 5B–5F). These low-density areas are apparent also in sections parallel to the plane of the membrane (Figures 5H–5K), which show that five discrete density peaks comprise one TspO monomer (circled in Figures 5H–5K), consistent with the prediction from hydropathy plots that each monomer contains five transmembrane helices. Each TspO dimer in the helical crystal is surrounded by a low-density area corresponding to the annular lipids, which is evident from Figures 5B–5F and 5H–5K. There are, of course, other possibilities for which a group of five density peaks in the dimer corresponds to the TspO monomer. Currently we favor the interpretation depicted in Figures 5H–5K because of its simplicity and how well it conforms to generally accepted principles of membrane protein structure. In addition, there appears to be no discernable density that could represent potential interhelical loops between the proposed monomers, which is also consistent with this interpretation.

### Interpretation of the Density Map

**Figure 6:**
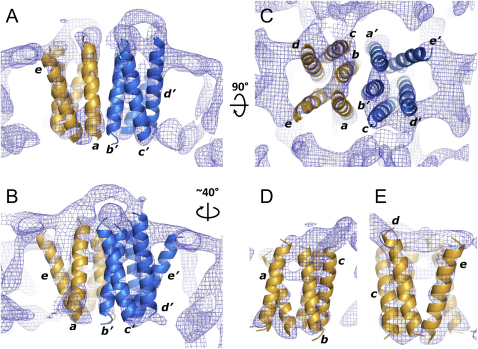
Interpretation of the Experimental Density Map (A) A view perpendicular to the membrane plane of the TpsO dimer with α helices fitted into the density map by eye. Red and blue α helices represent individual monomers of TspO and they are labeled arbitrarily *a–e* and *a′–e′*. (B) View parallel to the membrane plane of the TspO dimer and after a 40° rotation to show the highly tilted helices *e* and *e*′. (C) View perpendicular to the membrane plane. (D) TspO monomer viewed parallel to the membrane plane, from the perspective of the dimer interface formed by helices *a* and *b* and *a′* and *b′*. (E) TspO monomer viewed from the lipid bilayer. The experimental density maps in all the panels was contoured at 1.5 σ.

The low-resolution density map contained a number of rod-shaped densities that we have interpreted as transmembrane α helices. We have fitted α-helical backbones manually into these densities (Figures 6A–6E; Figure S4) either by modeling idealized α helices directly into the continuous density features or by connecting the strongest density elements within the map in a way that satisfied simple α-helical bundle geometry. At 10 Å resolution, it is not possible to assign the TspO sequence to the positions in the 3D map, so each putative transmembrane α helix is labeled *a*–*e* in one monomer and *a*′–*e*′ in the other monomer of the TspO dimer (Figure 6A). The interface between the two TspO monomers is formed by helices *a*, *b*, *a*′, and *b*′ (Figures 6A and 6D) and it is relatively flat without any large invaginations. Helices *b* and *b*′ of the two parallel monomers are the main structural elements that appear to be making intermonomer contacts throughout the lipid bilayer (Figures 6A and 6B). In contrast, a cleft is formed between helices *d* and *e* within the monomer, with helix *e* being the most highly tilted of all the helices (Figures 6C and 6E). Helix *c* forms contacts predominantly with helices *b* and *d*, although it may possibly form contacts with helix *a*′ in the adjacent monomer.

In addition to the transmembrane densities that we have interpreted as α helices, there are large areas of density at the ends of helices *c* and *e* that appear on the outside of the tubes (Figure 6; Figure S4). This density appears to form the major contact between adjacent dimers in the crystalline lattice and, in conjunction with the absence of an equivalent density on the opposite side of the membrane, it gives the dimer a distinctive wedge shape that defines the curvature of the tubes and contributes toward the overall helical crystal geometry. It is unclear at 10 Å resolution what the densities at the ends of helices *c* and *e* represent, but it is most likely to be the N terminus and/or C terminus, the latter still having the His_6_ tag attached. It seems less likely that the density represents a loop region between helices *d* and *e* given the large distance between the ends of these helices, but the additional masses at the ends of helices *a*–*c* probably do represent interhelical loops. However, contributions to all these membrane surface densities from ordered lipid head groups, or even perhaps porphyrin-like substrates copurified with the protein, cannot be wholly excluded.

## Discussion

Since its discovery as the “peripheral benzodiazepine receptor” in 1977 (Braestrup et al., 1977, Braestrup and Squires, 1977), TSPO has attracted considerable interest from both the pharmaceutical industry and academic research laboratories. High affinity drugs for the human homolog of the TSPO/PBR family have been developed for clinical applications and to elucidate its cellular functions, along with its involvement in physiological processes and pathophysiological conditions (Gavish et al., 1999, Papadopoulos et al., 2006a, Scarf et al., 2009). Indeed, much of what we have learnt about TSPO/PBR proteins was inferred from indirect pharmacological evidence (Gavish et al., 1999, Papadopoulos et al., 2006a). Only a few studies have attempted to address the structure of TSPO (Murail et al., 2008) and most of these have addressed the structural properties of drug binding sites as inferred from structure-activity assays (Anzini et al., 2001, Cinone et al., 2000). Consequently, up to now most of the questions regarding how the members of this protein family are organized on the molecular level remain unresolved. The low-resolution reconstruction presented here provides a first glimpse of the architecture of TspO in its native environment, the lipid bilayer.

TspO from *R. sphaeroides* was purified to homogeneity in DDM in a functional dimeric form, as suggested from the blue native PAGE analysis (Figure 2) and the cryo-EM structure of TspO (Figure 5). The observed dimeric form of TspO is consistent with mutagenesis data, which showed that the mutation W38C in TspO leads to the generation of covalent dimers due to the formation of an intermolecular disulphide bond (Yeliseev and Kaplan, 2000). Here, we show that TspO is probably a dimer in the membrane based on the 3D structure of TspO at 10 Å resolution determined from cryo-EM images of helical tubes. The repetitive unit in the tubular crystals is a bundle of five α helices, which is consistent with the proposal that the TspO monomer contains five transmembrane domains as predicted from hydropathy analyses of the primary amino acid sequence and confirmed by epitope mapping experiments (Joseph-Liauzun et al., 1998). The five α helices are packed into a ten helix bundle related by a two-fold symmetry axis perpendicular to the membrane. This dimer (Figure 6) is the most likely of all the possible dimers in the crystal structure to be of physiological relevance, due to the close proximity of the two monomers within the transmembrane region and the relatively large interface between them. Surrounding this dimer are regions in the crystal that show no strong density and are therefore predicted to contain lipid, although strong density is present at the predicted membrane bilayer surfaces, particularly on the outside face of the tube. The origin of this density is unclear, but it may represent ordered regions of either the N or C termini that mediate crystal contacts between neighboring TspO dimers. The densities for the transmembrane α helices are sometimes weaker than we might have anticipated, especially in the center of the lipid bilayer, as is seen for helices *a*, *b*, and *c*. These helices were modeled as continuous straight helices through the membrane, which is possible if the discontinuities in the density have arisen because of the low resolution of the data or high helix mobility. Another possibility that cannot be entirely discounted is that the helices contain regions that are non-helical as seen in many membrane protein structures (Gouaux and Mackinnon, 2005). Careful inspection of the density map with adjusted contour levels reveals presence of weak connecting density in those areas where discontinuities are observed, lending support to our interpretation of the map (see Figure S4). However, higher resolution will be required to fully understand the structure.

Presently, we cannot assign the amino acid sequence of TspO to particular α helices in our low-resolution model. In addition, there is insufficient unambiguous data to suggest the order of helix-helix connectivity in TspO, which makes building a model of TspO from our structure more difficult; the model built of EmrE from the 7.5 Å resolution cryo-EM structure was facilitated by the presence of clear connectivity between two of the transmembrane α helices (Fleishman et al., 2006). However, it is tempting to suggest that transmembrane segment 2 of TspO may be equivalent to the position of helix *b* in the reconstruction (Figure 6). This suggestion is based on the observation that residue W38 in the loop near the edge of the transmembrane helix 2 of TspO forms covalent dimers when mutated to cysteine (Yeliseev and Kaplan, 2000). Helices *b* and *b*′ in the reconstruction make the closest intermonomer contacts and it is conceivable that a crosslink between residues C38 of the two monomers may occur in this region.

The dimeric structure of TspO raises questions regarding the mechanism of transport of substrates across the membrane, the stoichiometry of substrate and inhibitor binding and where these bind in the transporter. In most structures of solute transporters that contain six or more α helices, there is one translocation pathway per polypeptide chain, regardless of whether the protein forms a monomer, trimer, or tetramer, although there are a few exceptions, e.g., Sav1866 (Dawson and Locher, 2006). Another example of a transporter where the binding site is formed at the interface between monomers is EmrE, which contains four transmembrane α helices per monomer, which combine to form an asymmetric homodimer; each dimer binds one molecule of substrate at the interface between the two monomers (Korkhov and Tate, 2009, Ma and Chang, 2007, Ubarretxena-Belandia et al., 2003, Ubarretxena-Belandia and Tate, 2004). A small molecule binding site is typically formed from a crevice in a bundle of helices. In the structure of the TspO dimer (Figure 6) the most likely substrate binding pocket is, therefore, between helices *d* and *e* and between *d*′ and *e*′,which implies that there are two binding sites per dimer. This crevice also appears to be relatively accessible from at least one leaflet of the lipid bilayer, which is characteristic of multidrug and lipid transporters like EmrE (Ubarretxena-Belandia et al., 2003), Sav1866 (Dawson and Locher, 2006), and P-glycoprotein (Aller et al., 2009). It is also consistent with the proposal that the TSPO family members can transport large hydrophobic compounds such as sterols and porphyrins (Papadopoulos et al., 2006a). The other possibility is that the binding site is at the monomer-monomer interface, as seen in EmrE, which would imply that one substrate molecule binds per dimer. However, this appears to be less likely because this interface in TspO appears relatively flat with the major interactions mediated by helix *b* from each monomer. It is, therefore, clearly desirable to determine the stoichiometry of substrate binding to either TspO or to one of its homologs, as this would allow the distinction between these two hypotheses. Similar data were important in defining the functional unit of EmrE as a homodimer (Butler et al., 2004), but this required an accurate radioligand binding assay, using a substrate that bound with high affinity, coupled to amino acid analysis of the purified protein. The lack of high-affinity radioligands for TspO precludes such a study in our work, although this may be possible for mammalian TSPO, which has a greater diversity of radiolabeled substrates and inhibitors, provided that TSPO can be overexpressed and purified in a fully functional form.

The low-resolution cryo-EM structure of TspO we present here is suggestive of two binding sites per dimer, which, in turn, allows for the possibility of cooperativity during substrate transport and potential effects of allosteric modulators. Given that human TSPO is 33.5% identical to *R. sphaeroides* TspO, it is likely that human TSPO will have a similar architecture to bacterial TspO, implying that human TSPO could also have two substrate translocation pathways and thus two potential drug binding sites in close proximity. Thus, the structure could explain the previously observed allosterism of ligand binding to TSPO/PBR proteins (Masonis and McCarthy, 1996) and the existence of several classes of drug binding sites (Boujrad et al., 1994, Boujrad et al., 1996). Experimental determination of the oligomeric state of human TSPO will therefore be extremely informative. The structure of *R. sphaeroides* TspO at higher resolution will help to define further the pathway of substrate translocation and will provide a valuable framework for future studies on this family of proteins.

## Experimental Procedures

### Reagents

Chemicals were purchased from Sigma-Aldrich. Hemin, PPIX, and cPPIII were purchased from Frontier Scientific. DDM (Anagrade) was purchased from Anatrace. *E. coli* polar lipids were purchased from Avanti Polar Lipids. TspO was isolated by PCR from plasmid pUI2701, generously provided by S. Kaplan, and cloned into plasmid pET29 (Novagen).

### Protein Expression and Purification

TspO was cloned into the NdeI and XhoI sites restriction sites of plasmid pET29 in *E. coli* strain BL21(DE3) for expression from the T7 promoter. Briefly, cells transformed with the pET29-TspO plasmid were grown to an OD_600_ of 2.0–2.5 in 2xTY medium and induced with 0.5 mM IPTG for 3 hr at 32°C. The cells were harvested by centrifugation (15 min, 5000 × g, 4°C). The pellets were resuspended in buffer A on ice [20 mM Tris (pH 7.5) and 200 mM NaCl] and the cells broken open using a continuous flow cell disruptor. After a brief spin at low speed (30 min, 10,000 × g, 4°C), the cell membranes were collected using a Ti45 rotor in an ultracentrifuge (2 hr, 170,000 × g, 4°C), resuspended in buffer A, flash frozen in liquid nitrogen, and stored at −80°C. For purification, the membranes were solubilized in buffer A supplemented with 2% DDM, and the insoluble material was removed by ultracentrifugation as before. The protein was purified using a His_6_ tag on NiNTA-agarose (QIAGEN), according to the manufacturer's protocol. The protein eluted from the Ni-affinity column was separated from the aggregates using gel filtration in 20 mM Tris (pH 7.5), 30 mM NaCl, and 0.025% DDM.

### Inner and Outer Membrane Separation

To separate the inner and outer membranes of *E. coli*, a two-step sucrose gradient fractionation protocol was used. The membrane preparations from TspO-expressing cells were mixed with sucrose (20% final concentration) and layered onto a pre-formed 70%–60% two-step sucrose gradient containing equivalent amounts of buffer and salt in a centrifuge tube (final volume ratio for the solutions with different sucrose concentrations of 1:1:1). Following centrifugation (16 hr, 260,000 × g, 4°C) the bands corresponding to inner membranes (golden layer between the 20% and 60% sucrose layers) and outer membranes (white layer between the 60% and 70% sucrose layers) were collected, diluted with buffer A, and centrifuged (2 hr, 310,000 × g, 4°C). The final pellet was resuspended in buffer A and used for SDS-PAGE and western blot analysis, according to standard procedures. For TspO-His_6_ detection, an HRP-conjugated His tag antibody (Novagen) was used, following the vendor's instructions.

### Blue Native PAGE

To determine the oligomeric state of the protein in detergent micelles, blue native PAGE was performed (Schagger and von Jagow, 1991). The 4%–16% gradient Novex Bis-Tris gels (Invitrogen) and the commercially available cathode and anode buffers for blue native PAGE (Invitrogen) were used. Protein samples in DDM were mixed with the loading buffer and loaded on a gel (5–10 μg protein/lane). Electrophoresis (performed at 4°C for 4–6 hr) and gel destaining were performed according to the vendor's instructions.

### Analytical Size Exclusion Chromatography

A purified TspO sample, concentrated to 1 mg/ml, was loaded on a Superdex 200 10/300 GL column, pre-equilibrated with buffer composed of 20 mM Tris (pH 7.5), 200 mM NaCl, and 0.025% DDM. The UV absorbance at 280 nm was monitored during chromatography and plotted against time (in minutes).

### Binding Assays

To assess the interactions between purified TspO and porphyrins, intrinsic tryptophan-fluorescence quenching was used. In brief, purified TspO in DDM at a final concentration of 2.5 μM was titrated with increasing amounts of heme in the desired concentration range. Each titration point was followed by a spectrum measurement (excitation at 295 nm, emission at 300–400 nm) and detection of tryptophan fluorescence. Arbitrary fluorescence units at 340 nm were plotted against ligand concentration to evaluate the fluorescence quenching. The apparent dissociation constants, K_app_, were estimated using a simple one-site binding model in GraphPad Prism.

### Crystallization of TspO Tubes

Reconstitution of the purified TspO into helical tubular crystals was performed in a setup similar to that used previously for EmrE (Korkhov and Tate, 2008, Tate et al., 2001). The protein preparations at final concentrations of 0.5–1 mg/ml were incubated with *E. coli* polar lipid extract at lipid/protein ratios of 0.2–0.6 in Slide-A-Lyzer cassettes (Pierce) submerged in 100 ml beakers filled with dialysis buffer [20 mM Tris (pH 7.5), 100 mM NaCl, and 2 mM EDTA] for 3 days, with daily exchange of dialysis buffer. The tubular crystals obtained this way were used directly for cryo-EM.

### Electron Microscopy

For freezing and cryo-EM, tubes of TspO at a concentration of 0.5 mg/ml in reconstitution buffer were pipetted on to a glow-discharged holey carbon grid (Agar Scientific). The solution was blotted from the opposite side of the grid with a strip of filter paper and the grid was plunged into a reservoir of liquid ethane. Grids frozen this way were then cryo-transferred into storage boxes. Standard cryo-transfer procedures were then used with a Gatan 626 cryo-stage for electron microscopy. The tubes were analyzed with a Tecnai F20 electron microscope operating at an accelerating voltage of 200 kV, using standard low-dose imaging conditions (10–15 e/Å^−2^). Images were acquired on film, using flood-beam illumination at a magnification of 50,000 with an underfocus between 0.7 and 2.3 μm. For further analysis, 36 micrographs were digitized using a home-built KZA scanner (Henderson et al., 2007) with a 6 m step size and processed as described below. For the hand determination, TspO tubes were negatively stained using a 0.75% uranyl formate solution (Ohi et al., 2004); we recorded images of these tubes on an FEI Tecnai T12 microscope at a magnification of 42,000 using a 2 × 2k CCD camera. The final pixel size on the specimen of 3.25 Å/pixel was calibrated by measuring the (23 Å)^−1^ layer line of 18 tobacco mosaic virus (TMV) reference specimens.

### Image Processing and 3D Image Reconstruction

The structure of TspO lipid tubes was determined using a recently developed single particle-based helical reconstruction algorithm (Sachse et al., 2007). Initially, the method was based on iterative helical real-space reconstruction (Egelman, 2007) but has been significantly extended by applying a full correction of the contrast-transfer function (CTF) of the microscope, imposing restraints on the alignment and a new symmetrization procedure (Sachse et al., 2007). This improved single-particle reconstruction procedure imposes helical symmetry in real space and thus requires precise knowledge of parameters of helical symmetry. Therefore, we derived initial parameters of helical symmetry from image analysis of the helical diffraction pattern of averaged class sums (Crowther et al., 1996) and further refined it using the single-particle method described below.

### Fourier-Bessel Analysis of Diffraction Pattern and Determination of Helical Symmetry Parameters

First, we inspected the helical diffraction pattern on single tubes. For this analysis, TspO lipid tubules were boxed, padded, and floated using Ximdisp (Smith, 1999) and were Fourier transformed. Selected tubes possessed three layer lines at (102 Å)^−1^, (48 Å)^−1^, and (32 Å)^−1^. Because of the limited signal present in single tube images, we classified the segmented tube stack using multivariate statistical analysis and further analyzed two well-ordered classes with apparent stacking striations from a total of twelve classes (Figure S1B). The remaining classes did not show clear stacking or moiré features owing to inherent crystal disorder and insufficient separation of different azimuthal or out-of-plane views. The amplitudes and phases of the best two classes could be readily analyzed and the helical lattice was indexed. The (32 Å)^−1^ layer line exhibits a maximum on the meridian and has, therefore, a Bessel order of 0. The reciprocal radius of the (102 Å)^−1^ and (48 Å)^−1^ layer line maxima and the presence of equal phases at the layer line peaks mirrored along the meridian indicate a Bessel order of 12 with opposite signs, respectively (Figure S1C). Hence, the derived lattice consists of helically related stacked rings, each containing a 12-fold rotational symmetry (Figure S1D). This configuration resulted in helical symmetry parameters of 9.6° and 32.4 Å. Because of the possible ambiguity in Bessel order determination, we also tested single-particle helical reconstructions with 10, 11, 13, and 14 start helical lattices, but none of these reconstructions were meaningful (data not shown). In contrast, the diffraction pattern and side views of the 12 start helical lattice model showed maximum correlation with the diffraction of the experimental image data and their class averages (data not shown). In addition, we also performed a classical Fourier-Bessel 3D reconstruction using three selected tubes with good diffraction (Crowther et al., 1996). The resulting 30 Å resolution map is comparable with the low-pass-filtered version of the single-particle reconstruction (data not shown).

### Refinement of Helical Symmetry Parameters

Optimization of helical symmetry parameters started at a helical rise of 32.4 Å and a helical rotation of 9.6°. The grid searches covered 25 combinations ranging from 32.0–32.8 Å in helical rise to 9.2–10.0° in helical rotation (Figures S1F and S1G). For each symmetry combination, four cycles was sufficient to produce a stable 3D reconstruction with each set of fixed symmetry parameters. We evaluated the cross-correlation between the power spectra sum of in-plane rotated segments and the simulated power spectrum of the 3D reconstruction of the particular combination of helical symmetry parameters. The optimal combination of helical symmetry parameters was determined by a grid search subdivided into a finer grid until the cross-correlation converged. In order to minimize the influence of noise and resolution-dependent amplitude scaling, we correlated resolution rings individually between 100 Å and 16 Å and used their average as a final measure (Figure S1G). Finally, a helical rise of 32.67 Å with a rotation of 9.53° produced maximum correlation between the sum of experimental power spectra and the simulated symmetry-imposed power spectrum of the 3D reconstruction (Figures S1D and S1E).

### Single-Particle Helical Reconstruction of TspO at 10 Å Resolution

TspO tubules of a width of ∼250 Å were segmented into overlapping images of 108 × 108 nm using Boxer from the EMAN software suite (Ludtke et al., 1999). We used a step size of 32 Å that directly corresponds to the repeat of the stacked rings. The CTF of the electron micrographs was determined using CTFFIND and subsequently refined by CTFTILT to yield segment-specific CTF parameters (Mindell and Grigorieff, 2003). Further image processing was performed using SPIDER software (Frank et al., 1996). For CTF correction, each segment was convoluted by the corresponding CTF and then subjected to iterative cycles of projection matching. Finally, angles around the helical axis were sampled in 2° intervals and out-of-plane tilt of the tubules was taken into account ±12° at 2° increments. Using the determined alignment parameters, each image segment was inserted 12 times into the 3D image reconstruction corresponding to the 12-fold rotational symmetry present in the ring. Asymmetric units related by the helical symmetry were already included as separate images on the stack due to the 32 Å step size used during the segmentation procedure. In addition to the helical lattice, we exploited the two-fold symmetry present within the TspO dimer. During projection matching, we found that there was no preferred polarity among the segments of a single tubule. Therefore, we aligned each segment twice with an in-plane rotation differing by 180° and included them in the 3D reconstruction. Finally, the volume was corrected by division of the average 3D value of CTF^2^ in addition to a Wiener filter constant of 1% of the maximum amplitude. In order to minimize noise, helical symmetry was also imposed on the final 3D volume after each cycle. We excluded segments whose (32 Å)^−1^ ring correlation of its power spectrum with the sum of all in-plane rotated power spectra was below 0.45. Because images were taken at low underfocus between 0.7 and 2.3 μm, the ring correlation at 32 Å is only minimally affected by CTF variation. Excluded segments with low ring cross-correlation showed weak layer-line diffraction. We included only 35%, or 2610, of the 7542 segments in the final 3D image reconstruction of 72 × 72 × 72 nm in size. The resolution of the final map was estimated using Fourier shell correlation criteria at 0.5/0.143 cut-offs yielding 10.2/7.8 Å, respectively (Figure S3). It should be noted that the numerical resolution cutoff appeared sensitive to the tightness of the applied mask. In order to avoid overestimation of the resolution, we dilated the structural mask by an additional 10 Å. The resolution estimate of 10.2 Å corresponds to the detail expected from a 10 Å resolution map. More image processing statistics are displayed in Table 1. The final volume was corrected for amplitude decay with a B factor of −400 Å^2^ while applying a figure-of-merit weighting scheme (Rosenthal and Henderson, 2003).

### Hand Determination

We determined the hand of TspO tubes using one-sided negative staining and helical diffraction analysis. Tubes from negatively stained grids showed significant asymmetry in their layer lines between adjacent quadrants in their power spectrum due to variation of stain thickness across the grid. Frequently, stain covers only the side of the particle facing the carbon (Klug, 1965, Klug and Finch, 1965). In the case of TMV, an asymmetric layer line was observed at 69 Å. According to the TMV reference structure (Finch, 1972), the (69 Å)^−1^ layer line corresponds to a Bessel order of 16 with left-handed helicity (Figure S2B). As expected, when imaging the specimen with the carbon side facing the electron gun, the striations present on the near side give rise to a stronger layer line on the right-hand side of the upper quadrant of the power spectrum (Figure S2C). In order to exclude asymmetric effects arising from different azimuthal views, we performed a control simulation, which shows that the (69 Å)^−1^ layer line is not subject to intensity variation during rotation around the helical axis or around the out-of-plane tilt axis (data not shown). The analysis of one-sided TspO tubules yields a (102 Å)^−1^ layer line, which shows a stronger reflection on the right-hand side of the upper quadrant, thus it also possesses left-handed helicity (Figures S2E and S2F). Therefore the (48 Å)^−1^ layer line carries the opposite sign according to the helical lattice. This asymmetry in the layer line is less obvious than for the (102 Å)^−1^ layer line. In order to increase the signal of the layer lines and compensate for long-range bending, overlapping segments of the tubes were excised using BOXER (Ludtke et al., 1999), rotated in the image plane, and Fourier transformed, and their power spectra were averaged. By analogy, we performed these tests for the structure of TspO and came to the same conclusion that the observed asymmetry in the (102 Å)^−1^ layer line is not due to different azimuthal or out-of-plane views. We also performed rotary shadowing analysis and tilting experiments to try and determine the hand of the structure independently; results for both methods were inconclusive. First, no lattice features were revealed by the smooth-surfaced helix in the platinum shadow. Second, we tested tilting the helix perpendicular to the helical axis (Finch, 1972), but, due to the relatively long helical pitch, insufficient asymmetry was shown by either the left or right side of the projections.

## Accession Numbers

The reconstructed TspO map has been deposited at the EMDB databank with accession number EMD-1698.

## Supplemental Information

Document S1. Four Figures
