# Supplementary material for: Three-Dimensional Structure of TspO by Electron Cryomicroscopy of Helical Crystals
Source: Structure. 2010 Jun 9;18(6):677–87. doi: 10.1016/j.str.2010.03.001 (PMC2911597; doi:10.1016/j.str.2010.03.001)
Supplement: Document S1. Four Figures [file mmc1.pdf]

Structure *18*

Supplemental Information

Three-Dimensional Structure of TspO

by Electron Cryomicroscopy of Helical Crystals

Vladimir M. Korkhov, Carsten Sachse, Judith M. Short, and Christopher G. Tate

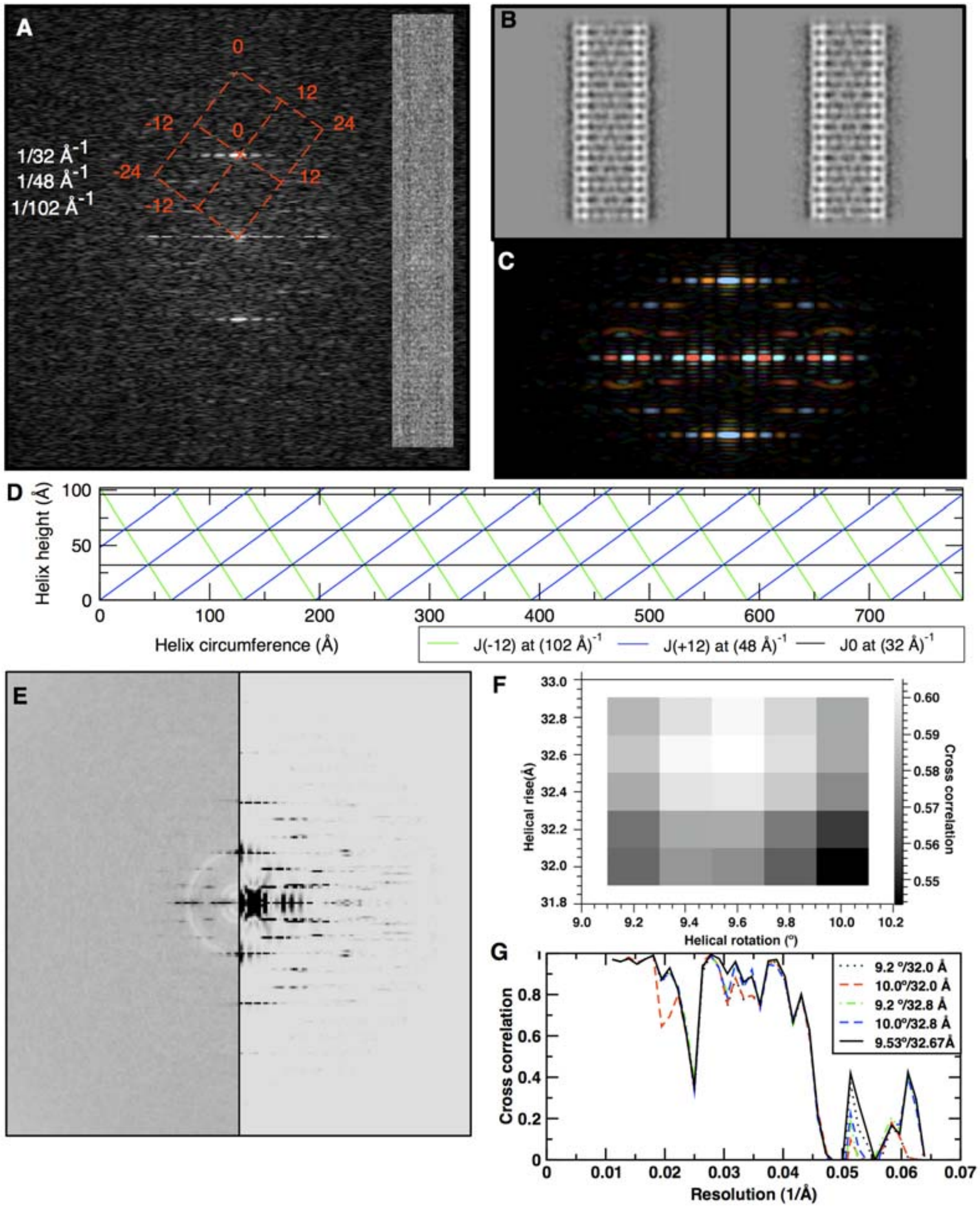

Figure S1. Determination of helical symmetry parameters
(A) Raw power spectrum of one well-preserved tube (right) overlaid with the helical lattice index, with the layer-line positions in white and the corresponding Bessel orders in red.
(B) Two out of twelve averaged classes with apparent stacking striations (C) Fourier transform of the class in the right-hand panel in (B). Phases are superimposed on

amplitudes with a colour scheme taken from Ximdisp (Smith, 1999), with the same colour indicating equal phases.
(D) A helical lattice was built assuming the assigned Bessel orders of the layer lines, from which we derived initial estimates of the helical symmetry parameters. Helical rotation and rise were refined by maximizing the cross correlation between experimental and simulated power spectra from independently calculated reconstructions of a set of symmetry parameters.
(E) Side-by-side display of experimental vs. simulated power spectra allowed a visual check of the calculated helical symmetry parameters.
(F) The symmetry grid from 9.2-10.0º helical rotation and 32.0 – 32.8 Å helical rise shows maximum correlation at 9.6º/32.6 Å (white box).
(G) Resolution-dependent cross correlation between (100 Å)$^{-1}$ and (15 Å)$^{-1}$ for a set of symmetry combinations from (E) and the final 9.53º/32.67 Å combination.

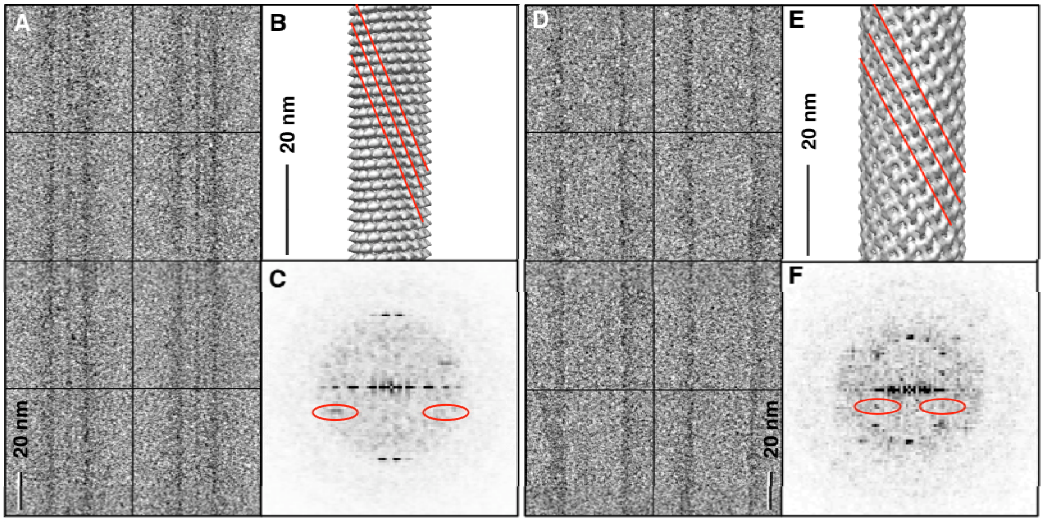

Figure S2. Determination of helix handedness by image analysis of one-sided negatively stained filaments
(A) Stack of overlapping TMV segments.
(B) Reference volume of TMV (Sachse et al., 2007) with highlighted left-handed helix of Bessel order n = -16 corresponding to the power spectrum (C), which shows the (69 Å)$^{-1}$ layer line (circled) with clear asymmetry in the upper right and lower left quadrant because of one-sided staining.
(D) Stack of overlapping TspO lipid tubes.
(E) Surface representation of the TspO 3D image reconstruction with highlighted left-handed helix of Bessel order n = -12 corresponding to the power spectrum in (F), which shows the (96 Å)$^{-1}$ layer line (circled) that possesses a stronger intensity maximum in the upper right and lower left quadrant compared with the upper left one.

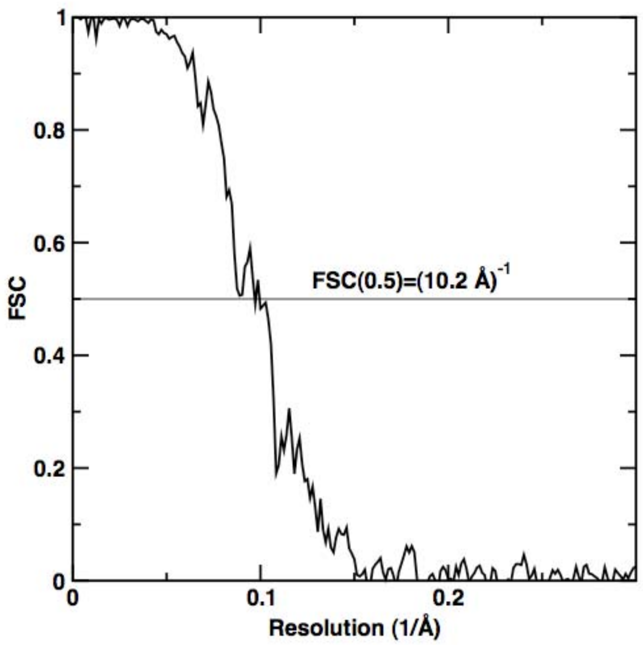

Figure S3. Resolution assessment for single-particle based helical reconstruction of TspO lipid tubes

The correlation was computed from two reconstructions containing half of the data set each, which indicates a resolution of either 10.2 Å or 7.8 Å according to whether the 0.5 or 0.143 cut-off criterion is applied.

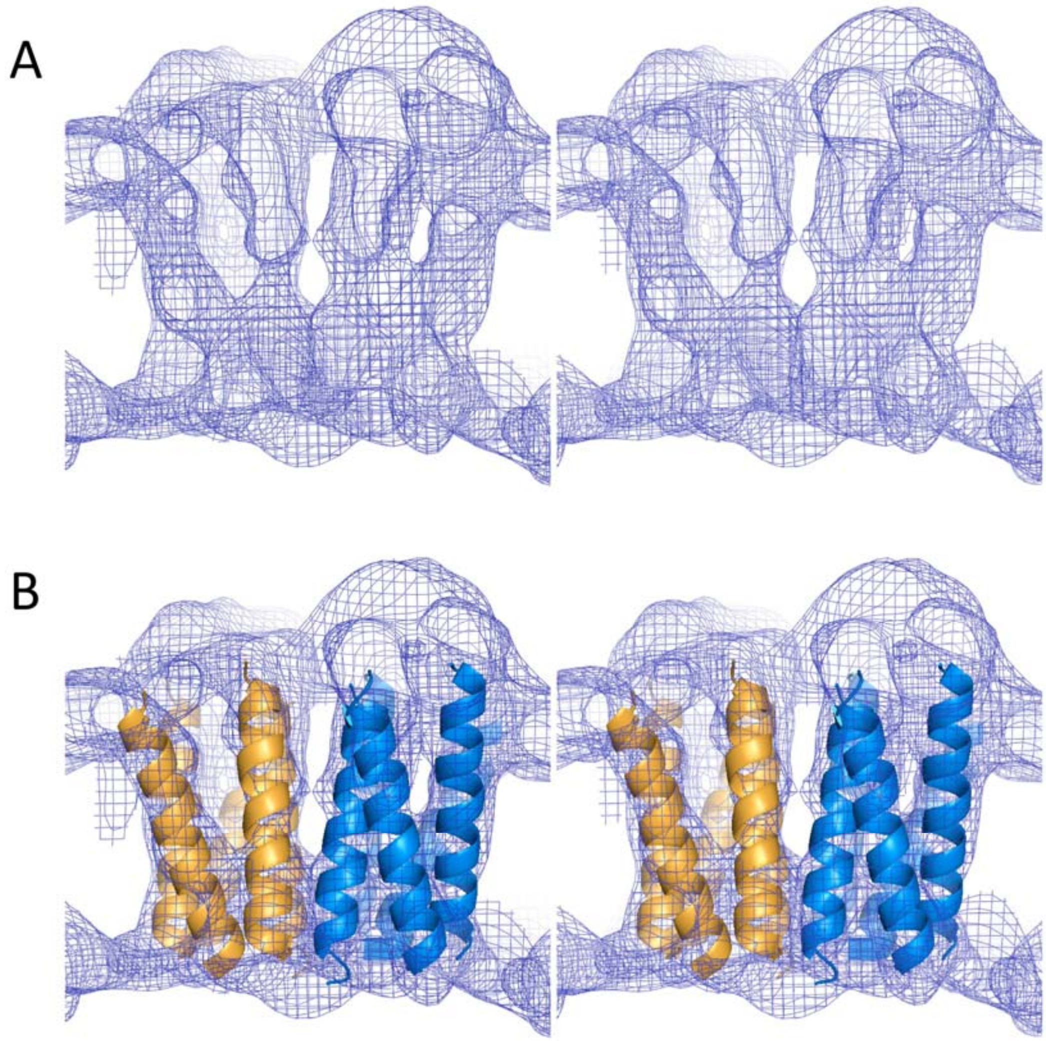

Figure S4. Stereo-images of the TspO density map
(A) A view of the density map parallel to the membrane plane, contoured at 1 σ.
(B) A density map stereo-view identical to (A), with modelled transmembrane helices in yellow and blue, corresponding to individual TspO monomers (as in Figure 6).
